# What the Destruction of the Voluntary Hospitals May Cost the State

**Published:** 1911-10-21

**Authors:** Godfrey H. Hamilton

**Affiliations:** National Hospital for the Paralysed and Epileptic.


### The Editor's Letter Box.

### WHAT THE DESTRUCTION OF THE VOLUNTARY HOSPITALS MAY COST THE STATE.

*To the Editor of* The Hospital.

Sir,—In your last issue Sir Henry Burdett is reported as saying: "Hospitals in this country require from £10,000,000 to £15,000,000 annually." Can you kindly tell me how this figure is arrived at, as I am in need of information as to the approximate annual cost for some work I am engaged on at present? From the "Annual" I make the annual expenditure for the British Isles much less than Sir Henry.—Yours faithfully,

October 10. Godfrey H. Hamilton.
National Hospital
for the Paralysed and Epileptic.

[Mr. Hamilton will find the information he seeks on page 71 of the present issue.—Ed., The Hospital.]

